# Whole-genome sequencing enhances existing pathogen and antimicrobial-resistance surveillance schemes within a neonatal unit

**DOI:** 10.1099/mgen.0.000841

**Published:** 2022-06-13

**Authors:** Vivien Price, Steven J. Dunn, Robert A. Moran, Jonathan Swindells, Alan McNally

**Affiliations:** ^1^​ Institute of Microbiology and Infection, College of Medical and Dental Sciences, University of Birmingham, Edgbaston, Birmingham B15 2TT, UK; ^2^​ Black Country Pathology Service, Birmingham City Hospital, Dudley Road, Birmingham, West Midlands B18 7QH, UK

**Keywords:** AMR, Gram-negative, infection control, neonatal, screening, transmission

## Abstract

In some neonatal units, the screening of isolates for antimicrobial-resistant organisms is a matter of routine, with theoretical benefits including the prevention or early detection of outbreaks. This study sought to use whole-genome sequencing (WGS) retrospectively to characterize the genomic epidemiology of Gram-negative organisms obtained from a screening programme in a 32-bed unit providing intensive, high-dependency and special care at City Hospital, Birmingham, UK, identifying occult transmission events and clinically important antimicrobial-resistance (AMR) genes. WGS was performed for 155 isolates collected from rectal and umbilical screening swabs over a 2 month period from 44 individual neonates. Genomic epidemiological analysis showed possible transmission events involving *Escherichia coli *, *Enterobacter cloacae *, *Klebsiella oxytoca* and *Klebsiella pneumoniae* not detected by routine screening, with eight putative clusters involving different individuals identified. Within phylogenetic clusters, the relatedness of organisms – as determined by the abundance of SNPs – varied widely, indicating that a variety of transmission routes may be implicated. While clinically important AMR genes were not present in the putative transmission clusters, our observation of suspected interspecies horizontal transfer of *bla*_CTX-M-15_ within individuals highlights the potential for their spread between organisms as well as individuals in this environment, with implications for surveillance. Our data show that WGS may reveal occult Gram-negative transmission events, demonstrating the potential of sequencing-based surveillance systems for nosocomial pathogens. Challenges remain in understanding how to utilize WGS surveillance to maximum effect in real-world settings.

## Data Summary

Sequence data is available from the National Center for Biotechnology Information under BioProject accession number PRJNA778615.

**Impact Statement**

Neonatal units care for the most unwell newborn babies, many of whom are treated for infection. There is growing concern that the bacteria that cause these infections are becoming resistant to antibiotics. Some neonatal units test babies for different bacteria as part of routine care – regardless of whether they currently have an infection or not – and use this information to detect outbreaks, but the pros and cons have not been well studied. We looked at bacteria from screening swabs taken from babies in a neonatal unit. We examined the genomes of these bacteria, and determined that some of them were very closely related, and had likely been passed from baby to baby during their time on the unit. We found eight clusters of organisms that were suspicious for transmission between babies, as well as evidence of resistance gene transfer between different bacterial species within the same baby. This has important implications for surveillance of infection in this patient group. This study shows the potential of whole-genome sequencing surveillance to detect transmission of bacteria between babies that would otherwise have gone undetected. Challenges remain in understanding how this technology can best be applied in routine practice for surveillance of bacteria causing hospital-associated infections.

## Introduction

Neonatal units care for the most unwell newborn infants, who are highly vulnerable to infection [1]. Nosocomial outbreaks of a range of pathogens have been reported, and instances of outbreaks by antimicrobial-resistant organisms are of particular concern [1–4]. While screening for meticillin-resistant *Staphylococcus aureus* (MRSA) colonization is widely established, the additional practice of routine screening for antimicrobial-resistant Gram-negative organisms in faeces, stool or skin surface swabs has only been implemented by approximately one in five neonatal units in the UK [5]. This screening is usually targeted at organisms with extended-spectrum β-lactamase (ESBL) and/or gentamicin resistance. The theoretical benefits are twofold, the first being the prevention and early detection of outbreaks by informing infection prevention and control actions such as isolating and cohorting, and the second being to predict the causative organism in subsequent instances of clinically suspected sepsis, whilst also guiding antibiotic therapy by targeting antimicrobial-resistant isolates from colonization screens. There is, however, a paucity of evidence for the purported benefits and, therefore, routine use is not recommended by national guidance in the UK [6].

Effects of routine Gram-negative screening on infection prevention and control initiatives can be difficult to measure. Much of the literature in this area focuses on the investigation and management of outbreaks [7] rather than interrogating the performance of screening as prospective surveillance. The impact of colonization in helpfully guiding management of clinical infection is also tenuous. While Gram-negative colonization may be found in approximately 50 % of neonates [8], the rate of subsequent invasive clinical infection is very low, and the usefulness of using these results to guide empiric antibiotic therapy is unclear. One paper reported screening 4449 individuals in order to predict one episode of Gram-negative sepsis with a gentamicin-resistant organism [9]. Furthermore, a small proportion of isolates with phenotypic susceptibility do carry resistance genes [10], the significance of which is not clear.

At Birmingham City Hospital (part of Sandwell and West Birmingham Hospitals NHS Trust, UK), Gram-negative screening in the neonatal unit has been routine for over 10 years. Every individual is screened on admission to the neonatal unit and weekly thereafter for the presence of Gram-negative organisms with resistance to gentamicin, and, in the case of *Escherichia coli* and *Klebsiella pneumoniae *, for the presence of ESBLs. This study aims to build on this screening strategy by using whole-genome sequencing (WGS) to uncover the genomic epidemiology of Gram-negative organisms isolated in the neonatal unit. By using a WGS approach, we are able to identify possible occult transmission events, as well as the presence or absence of clinically relevant antimicrobial resistance (AMR). This aims to inform decisions about the usefulness of screening in guiding infection prevention and control initiatives in this context.

## Methods

### Study aim, design and setting

The aim of our study was to evaluate the role of WGS in enhancing Gram-negative screening within the neonatal unit. The setting is a 32-bed neonatal unit providing intensive, high-dependency and special care to neonates. Routine screening for Gram-negative colonization was well established and had been ongoing for a period of more than 10 years at the time of data collection. Isolates were collected over a 2 month period from 2nd August 2019 to 23rd September 2019. As this study evaluates aspects of routine care, this was approved as a Service Evaluation by the Sandwell and West Birmingham Hospitals NHS Trust.

### Sample collection and selection of isolates for sequencing

Screening samples were collected in the form of swabs of all individuals on admission to the unit, and weekly thereafter as part of routine clinical care. Two swabs were taken for culture of Gram-negative growth, one from either rectum or stool, and one from the umbilicus. This was done as part of well-established routine screening programme for Gram-negative organisms, ongoing in this unit for at least 10 years, and performed alongside screening for MRSA and group B streptococci, which are not considered here. Swabs were plated onto a chromogenic media (Brilliance UTI media; Oxoid) and incubated at 37 °C overnight. *E. coli* was identified macroscopically on the basis of colour change and colony morphology, while all other colony types were identified using a MS system (bioMérieux) with a cut-off value of 2.0. Isolates of *Enterobacterales* were tested for susceptibility to cefpodoxime (as an indicator of likely ESBL carriage) and gentamicin (* E. coli* or *Klebsiella* species), or gentamicin alone, by the EUCAST (European Committee on Antimicrobial Susceptibility Testing) disc diffusion method [11]. Organisms resistant to these antibiotics were subjected to further antimicrobial-susceptibility testing using the Vitek platform (bioMérieux). Isolates are reported clinically if demonstrating resistance to cefpodoxime or gentamicin, or if an organism of potential high risk of environmental acquisition was isolated (e.g. *Pseudomonas aeruginosa *). This was performed as part of the standard workflow of the clinical laboratory. As this was a study evaluating swabs collected as part of routine care, environmental samples were not collected.

All samples from which Gram-negative growth was macroscopically evident were included. For this study, single colony picks of all phenotypically distinct confirmed or suspected Gram-negative isolates were collected from chromogenic plates prospectively. These were sub-cultured onto chromogenic media (UTI ChromoSelect media; Sigma-Aldrich) and incubated overnight. They were examined macroscopically to confirm pure growth prior to storage at −80 °C.

Isolates of the most prevalent species – *E. coli *, *Enterobacter cloacae* complex, *Klebsiella oxytoca* and *K. pneumoniae* – were selected for WGS. These species were chosen as they were most commonly isolated from routine culture and, therefore, most likely to enrich for transmission events due to their relative abundance across a number of individuals. Where the same individual had more than one isolate of the same species isolated from the same site on the same day, reflecting colony variation, antimicrobial-susceptibility testing was performed and antibiograms compared. Where antibiograms were discordant both isolates were selected for sequencing, otherwise one isolate only was selected. If the isolates came from different sites (i.e. umbilical and rectal swab) then both were selected, and if multiple species were isolated from the same sample then all were selected.

Gestational age at birth, admission and discharge dates, length of stay, respiratory support, nutritional support, and clinical diagnosis of necrotizing enterocolitis were collected from clinical records retrospectively for all individuals from whom isolates were submitted for WGS.

### Sequencing and genomic analysis

WGS was performed by MicrobesNG (http://microbesng.uk) using the Illumina HiSeq platform. Sequence reads were trimmed using Trimmomatic (version 0.3) [12], assembled using SPAdes with the --careful flag, and otherwise under default settings (version 3.313.0) [13]. The median sequencing coverage across the dataset was 85. The median number of contigs across all assemblies was 89, and median N50 was 525 073. All assemblies included in this study were within the expected genome size and G+C content (* E. coli* 4.6–5.4 Mb, 50.8 mol% G+C; *Klebsiella* spp. 5.1–5.9 Mb, 57 mol% G+C; *Enterobacter* 4.5–5.3 Mb, 55.5 mol% G+C). Assemblies were annotated using Prokka (version 1.1) [14] under default settings.

Species identification was provided by the MicrobesNG’s analysis pipeline, which uses Kraken [15]. Species were further confirmed and sequence type designations conferred using MLST (version 2.15; https://github.com/tseemann/mlst), and species-specific phylogenies, showing all members of the same species clustering together. AMR genes were detected using Abricate using the National Center for Biotechnology Information (NCBI) AMRFinderPlus database (version 0.8.7; https://github.com/tseemann/abricate). Phylogenies were initially reconstructed using Mashtree (version 2) [16], and visually inspected for clusters and AMR genes of importance using Phandango [17]. Isolates of the same species and sequence type or isolates appearing to cluster together on visual inspection of a phylogenetic tree were assessed using Snippy (v 4.3.6; https://github.com/tseemann/snippy) on standard settings, using annotated assemblies as references to allow inference on SNP location and amino acid change. The annotated assembly from the isolate with the earliest sample collection date was chosen as the reference, in order to map changes to similar isolate assemblies sampled later from the same individual or from other individuals, in order to enable inference of changes over time and possible or likely transmission. Assembly metrics within clusters were highly similar (N50±71 598, no. of contigs ±9). The Snippy-core function and Snp-dists (v 0.6.3; https://github.com/tseemann/snp-dists) were then used to assess relatedness in terms of SNP distances between isolates and used to infer amino acid changes. A SNP difference of up to 50 was used to infer possible transmission. This number was arbitrarily chosen; however, we aimed to capture diverse transmission methods including environmental sources with potential for significant genetic variability (e.g. organisms in environmental biofilms), and allow some mitigation for the incomplete reference sequences we were using.

**Fig. 1.**
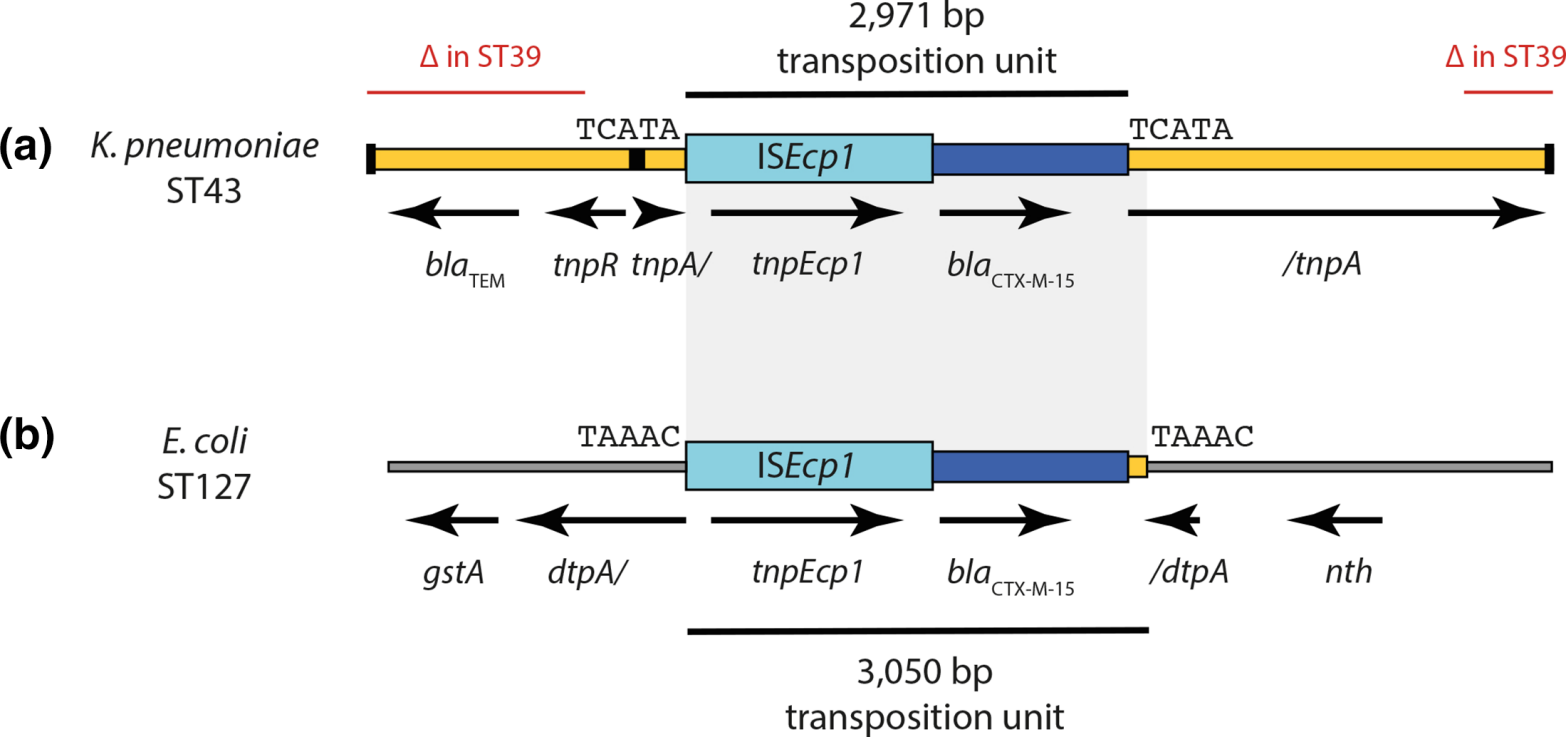
Contexts of *bla*_CTX-M-15_ in neonatal *K. pneumoniae* (a) and *E. coli* (b) isolates. DNA sequences are shown as scaled; coloured boxes – orange for Tn*2*, light blue for IS*Ecp1*, dark blue for the *bla*_CTX-M-15_-containing transposition unit passenger sequence, and grey for *E. coli* chromosome. The names and extents of genes are indicated below the boxes. The terminal inverted repeats and *res* site of Tn*2* are shown as black vertical lines at the end of the sequence and as a black box, respectively. Grey shading between parts (a) and (b) links identical DNA sequences. The size and extent of IS*Ecp1* transposition units are indicated above (a) and below (b), while the sequences of target site duplications generated by their insertion are shown on either side of them. The extent of truncations mediated by IS*26* in *K. pneumoniae* ST39 isolates are indicated by red lines labelled ‘∆ in ST39’ in (a).

Annotated assemblies of clustered isolates were inspected in Artemis, and searches were performed using the Basic Local Alignment Search Tool (blast) on the National Center for Biotechnology Information database (https://blast.ncbi.nlm.nih.gov/Blast.cgi) to elucidate the genes in which SNPs were observed. IS*Ecp1* transposition units were annotated manually in Gene Construction Kit v4.5 (Textco Biosoftware) and compared using blast. Scaled representations of these sequences were exported to Adobe Illustrator v25.4.1 to generate Fig. 1.

## Results

### Species identification by WGS reveals some discordance with phenotypic methods used in the diagnostic laboratory

**Fig. 2.**
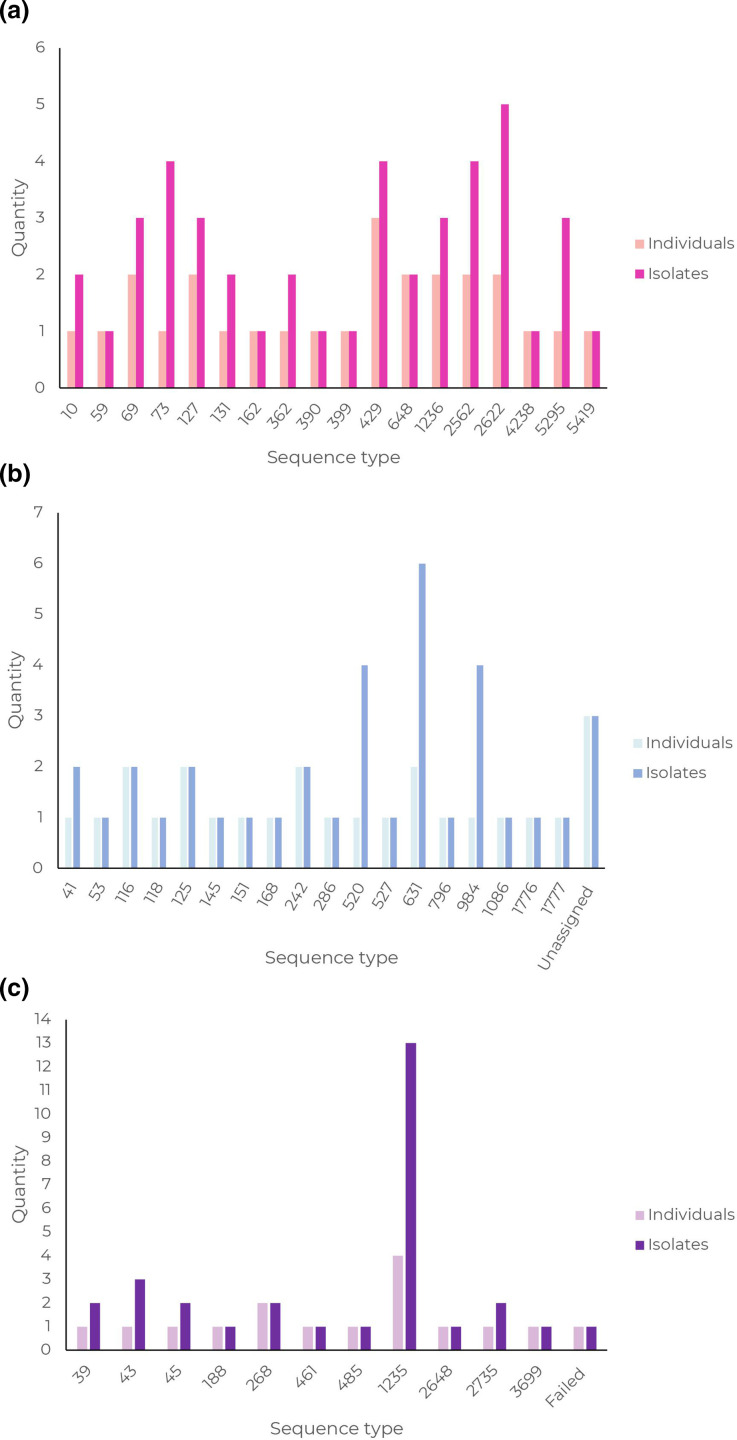
Sequence type by MLST for (a) *E. coli *, (b) *Enterobacter cloacae* and (c) *K. pneumoniae *, shown by frequency of unique individuals colonized (series 1) and total number of isolates per sequence type (series 2). *K. oxytoca* sequence types are not pictured graphically due to the very low number for which a sequence type could be ascribed.

Over a 2 month period in August and September 2019, all isolates obtained from routine neonatal unit surveillance samples were collected. These samples represent admission and weekly screening samples from faecal/rectal and umbilical sampling, and comprised 261 isolates from 111 samples, obtained from 48 individuals (Fig. S1, available with the online version of this article), of which 155 isolates from 44 individuals underwent WGS. The majority (*n*=35) of neonates were admitted to the unit on the day of birth, 3 on the day following birth, and the remainder between 3 and 17 days post-birth. It should be noted that many, if not all, of these cases will represent transfers from another neonatal unit of a baby that has never lived in the community, although this is not captured by our data. Of these, we identified 43 *E. coli *, 38 *Enterobacter cloacae *, 35 *K *. *oxytoca *, 30 *K *. *pneumoniae *, 4 *Cronobacter* species, 3 *Klebsiella aerogenes* and 1 *Citrobacter freundii* (Fig. 2a), and 1 isolate failed data extraction. The species identified by WGS was discordant with the identification provided by MS in 13 instances. All cases of discordant identification were between members of the *Enterobacterales *.

Sequence types of the most prevalent organisms are shown in Fig. 2). Eighteen different sequence types of *E. coli* were identified. The most prevalent sequence types were ST429 (three individuals, 4 isolates), ST2622 (two individuals, 5 isolates), ST2562 (two individuals, 4 isolates), ST69 (two individuals, 3 isolates), ST127 (two individuals, 3 isolates) and ST1236 (two individuals, 3 isolates). For *Enterobacter cloacae *, 16 different sequence types were identified, including 2 novel sequence types designated as ST1776 and ST1777. One isolate could not be assigned a sequence type due to absence of the *rpoB* gene, and two due to sequence quality. The most prevalent sequence types were ST631 (two individuals, 6 isolates), and ST116, ST125 and ST242 (all with two individuals, 2 isolates). For *K. oxytoca *, sequence types could only be ascribed for four isolates, in keeping with the relative underdevelopment of the multilocus sequence typing (MLST) scheme for this organism. In both instances, one individual had two isolates of sequence types ST2 and ST92, respectively. The remaining *K. oxytoca* isolates have been submitted to the database curator for consideration of novel sequence type designation, the outcome of which is awaited at the time of writing. For *K. pneumoniae *, 11 different sequence types were identified by MLST, with the most prevalent sequence type being ST1235 (four babies, 12 isolates), followed by ST268 (two individuals, 2 isolates).

Within this dataset were four isolates of *Cronobacter sakazakii* from four different individuals, all of which had previously been identified as other members of the *Enterobacterales* by the diagnostic laboratory. The three previously believed to be *Enterobacter cloacae* had been identified using MS on the bioMérieux MALDI-TOF platform, while the isolate previously believed to be *E. coli* had been identified macroscopically on the basis of pink colour change on chromogenic media, in line with standard operating procedures. MLST identified sequence types for three of the four isolates, with two belonging to ST420 and one to ST6 of species *Cronobacter sakazakii *. In the MLST database, ST420 has previously been isolated from oat flakes [18], and ST6 has been isolated from baby formula [19, 20]. The two ST420 isolates were not closely related, being separated by 17 521 SNPs.

### Identification of putative transmission clusters within the sampled isolates

**Fig. 3.**
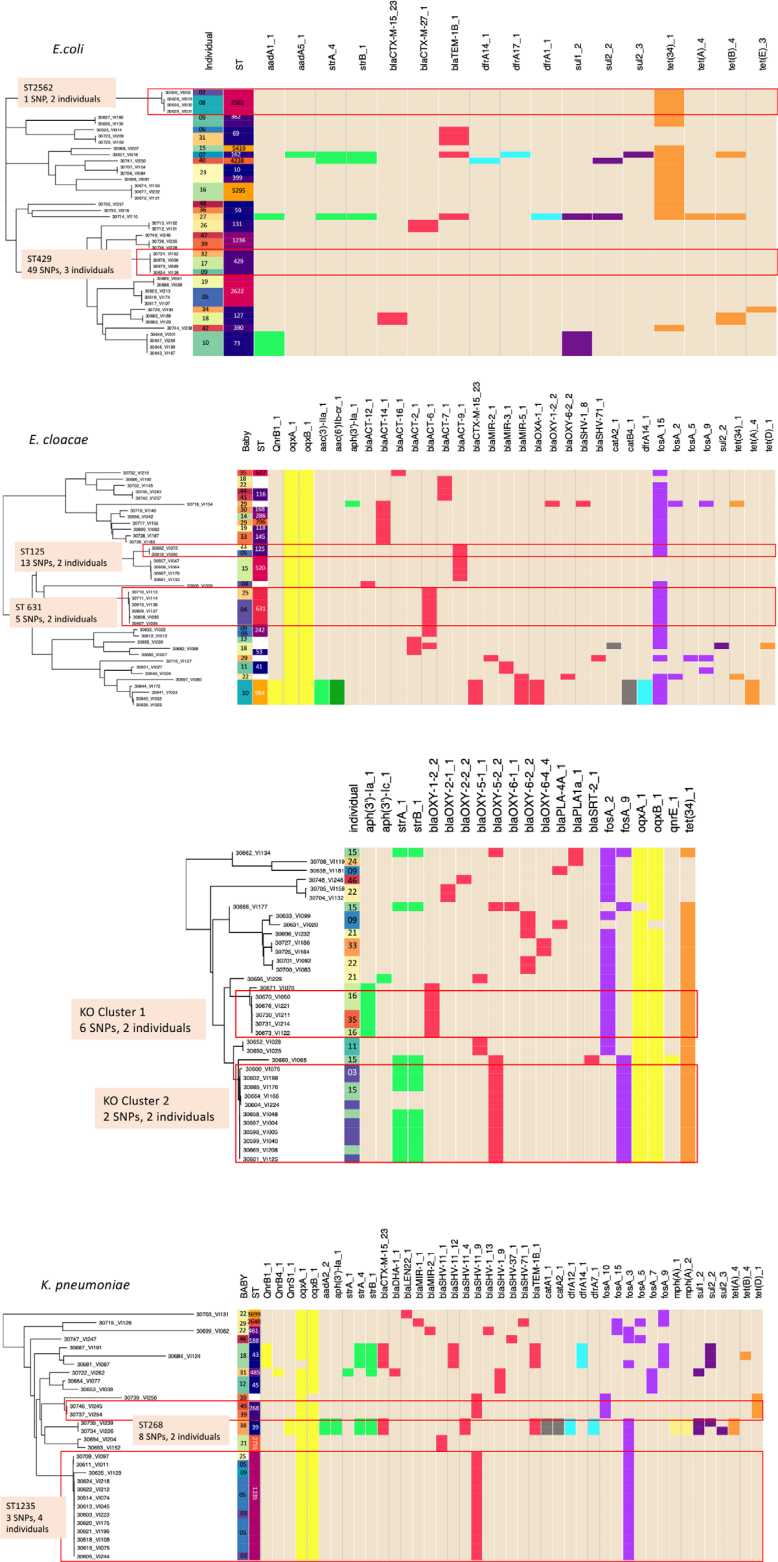
Broad overview of sequenced isolates shown as phylogenetic trees for *E. coli *, *Enterobacter cloacae *, *K. oxytoca* and *K. pneumoniae *, demonstrating the breadth and distribution of sequence types and AMR genes present across the different individuals on the unit. Overlaid are details of sequence types and participant numbers. More detailed views of the putative transmission clusters are presented in Figs 4 and S2. The red rectangles indicate putative transmission clusters. Clusters occurred across all species types, but did not contain any AMRs deemed to be of clinical significance. Genes are colour coded by their resistance to antibiotic classes: red, β-lactams; bright yellow, quinolone; pale yellow, macrolide; bright green, aminoglycoside; dark green, aminoglycoside and quinolone; orange, tetracycline; dark purple, sulphonamide; light purple, fosfomycin; light blue, trimethoprim; grey, chloramphenicol.

**Fig. 4.**
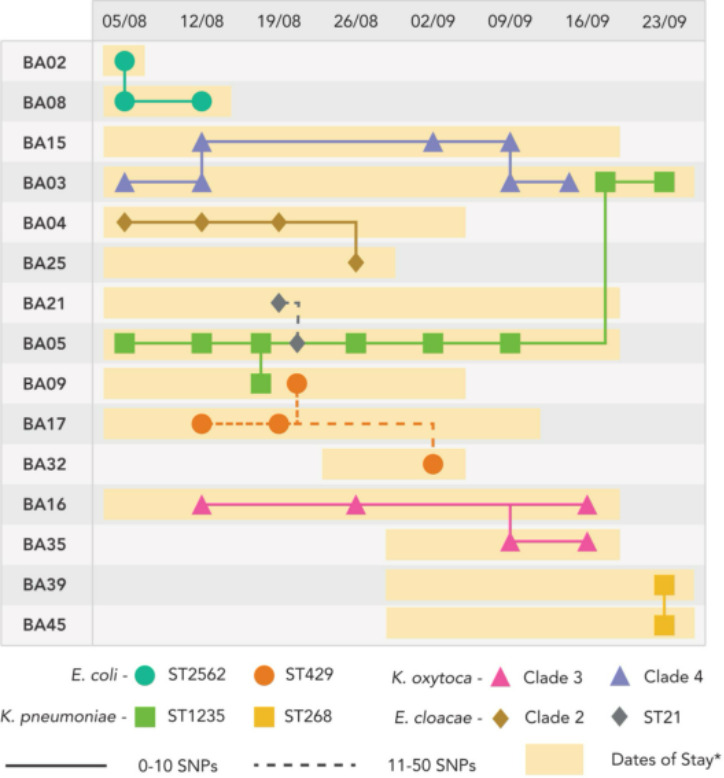
Putative transmission clusters occurring across different individuals across the study period. Clusters involved up to four individuals, and some individuals were involved in more than one cluster. The *y*-axis indicates individuals, numbered sequentially in order of enrolment. The *x*-axis represents dates in 2019 in dd/mm format. The shaded areas reflect duration of inpatient stay. Dates of stay reflect the week in which babies were admitted to the Neonatal Intensive Care Unit (NICU) (i.e. the start of the coloured range), and the week in which they were discharged (i.e. the end of coloured range).

We defined a putative cluster to be two or more isolates derived from two or more individuals with a SNP difference of 50 or less. Neonates admitted to the unit are usually admitted on the day of birth or within a short number of days thereafter. They would not be expected to ever have lived in the community, meaning that their flora must be acquired from the healthcare environment and people therein, including parents and hospital staff. Eight putative transmission clusters were identified, which are shown in broad overview in Fig. 3. These occurred across all four of the species studied and included 2–4 individuals. Additional phylogenetic trees showing each of the clusters in more detail, generated with Parsnp [21], can be seen in Fig. S2. The unit-wide distribution of putative clusters across different individuals, bacterial species and sequence types is show in Fig. 4. Overlapping admission dates were seen in all instances for individuals with isolates identified as being in a putative transmission cluster.

**Fig. 5.**
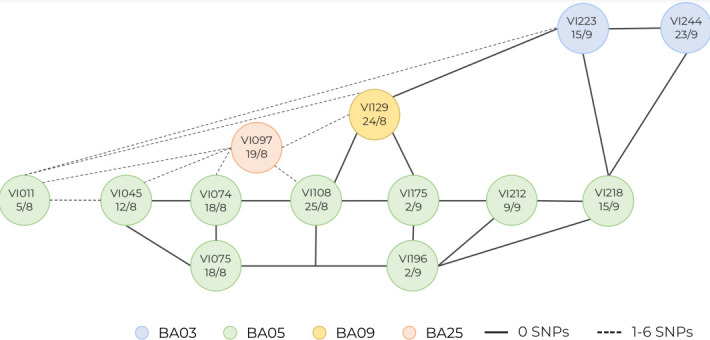
*
K. pneumoniae* ST1235 transmission cluster, showing transmission of a highly conserved strain between four individuals sampled over a number of weeks. Circles represent samples, with sample identification number and sample date (dd/mm, all 2019) indicated within, colours within circles represent different individuals (labelled sequentially in order of enrolment), and SNP differences are shown by solid lines (0 SNPs) or dotted lines (1–6 SNPs).

The largest putative cluster involved four individuals colonized with *K. pneumoniae* ST1235 (Fig. 5). This cluster consisted of 13 isolates separated by six or fewer SNPs. When compared to the earliest isolate as a reference, the organisms shared a common non-synonymous mutation changing amino acid residue 152 from asparagine to tyrosine in the *Bm3R1* gene, suggesting common evolutionary history. A cluster of two isolates from two individuals of *K. pneumoniae* ST268 were separated by seven SNPs. Two clusters were seen involving *K. oxytoca* isolates, neither of which were a known sequence type; therefore, they are described here as KO cluster 1 and KO cluster 2. KO cluster 1 involved five isolates from two individuals separated by 0–6 SNPs. KO cluster 2 involved 11 isolates from two individuals, separated by 0–2 SNPs. Isolates from both individuals had a splice variant with premature stop codon in the *epsJ* gene encoding a glycosyltransferase family 2 protein.

For *Enterobacter cloacae *, two clusters were observed, in ST125 and ST631. The ST631 cluster consisted of six isolates from two babies separated by 0–4 SNPs. The ST125 cluster included just two isolates from two individual babies, which were separated by 11 SNPs. In *E. coli *, a cluster was seen of ST2562 involving four isolates from two individuals separated by 14 SNPs. In *E coli* ST429, 19–44 SNPs distinguished four isolates from three babies.

### Clinically relevant AMR is rare but shows evidence of horizontal gene transfer (HGT) of resistance genes within the hospital unit

No ESBL or gentamicin-resistant phenotypes were observed within the putative transmission clusters; however, the ESBL-encoding gene *bla*_CTX-M-15_ was found in isolates of *E. coli* ST127, *Enterobacter cloacae* ST984, and *K. pneumoniae* ST39 and ST43. *E. coli* ST131 isolates from one individual carried the ESBL-encoding gene *bla*_CTX-M-27_. Carriage of a *bla*_CTX-M_ gene was associated with an ESBL phenotype in all but one case; three isolates of *bla*_CTX-M-15_-containing *K. pneumoniae* ST43 from a single individual were phenotypically sensitive to the cefpodoxime used for initial ESBL screening and, therefore, were not subject to further phenotypic testing. The *bla*_CTX-M-15_-containing *Enterobacter cloacae* ST984 isolates were also resistant to gentamicin, owing to the presence of the aminoglycoside-resistance gene *aacC2a*.

As *bla*_CTX-M-15_ was found in isolates of three different species over the course of this study, its genetic context in each isolate was examined to determine whether it could have been transferred horizontally between them. In *K. pneumoniae* ST43 isolates, *bla*_CTX-M-15_ was found in a 2917 bp IS*Ecp1* transposition unit inserted in transposon Tn*2* and flanked by the 5 bp target site duplication TCATA (Fig. 1a). Using the sequence shown in Fig. 1(a) to query the GenBank non-redundant nucleotide database revealed that this configuration is internationally disseminated and has been circulating since at least 2010 in plasmids in *Klebsiella variicola *, *K. pneumoniae *, *K. oxytoca *, *E. coli *, *Salmonella enterica* serovar Typhi and *Providencia stuartii* (Table S1). The *bla*_CTX-M-15_ gene was found in the same context in *K. pneumoniae* ST39 isolates, but there the left and right ends of Tn*2* have been truncated by IS*26* (extents of deletions marked in Fig. 1a).

In *E. coli *, ST127 *bla*_CTX-M-15_ is found in a 3050 bp IS*Ecp1* transposition unit inserted in the chromosome, interrupting the *dtpA* peptide transporter gene and flanked by the 5 bp target site duplication TAAAC (Fig. 1b). The characteristic transposition unit–chromosome junctions generated by this ST127 strain’s acquisition of *bla*_CTX-M-15_ are not found in GenBank (last search September 30 2021). The terminal 79 bp of this transposition unit are derived from Tn*2*, a configuration that can only have arisen following transposition from the context seen here in *K. pneumoniae* ST43 (Fig. 1b). Given the *bla*_CTX-M-15_-containing *E. coli* ST127 and *K. pneumoniae* ST43 were isolated from the same individual (baby 18), it seems highly probable that this ESBL-encoding gene has transferred from *K. pneumoniae* to *E. coli* in this setting.

The *bla*_CTX-M-15_ in *Enterobacter cloacae* ST984 was likewise found in a seemingly unique context in its chromosome. The *bla*_CTX-M-15_ gene in *Enterobacter cloacae* ST984 is adjacent to IS*Ecp1*. It, therefore, appears to have inserted into the *Enterobacter cloacae* chromosome as part of an IS*Ecp1* transposition unit. Downstream of *bla*_CTX-M-15_ are 2246 bp of Tn*2* and a copy of IS*26* in the same position seen in *K. pneumoniae* ST39. However, as the contig containing this sequence has broken in the repetitive IS*26* sequence, the extent of this transposition unit could not be determined from short-read sequence data.

Of the four individuals from whom organisms with *bla*_CTX-M_-mediated β-lactam resistance were detected phenotypically by the clinical laboratory, only one individual could be identified as having been cared for in isolation. For two individuals, there was clear documentation that care in isolation had not been possible for clinical reasons, and for one individual there was no information available regarding isolation.

Having identified possible transmission occurring within the unit, we attempted to identify any potential common environmental sources. We examined all metadata available to us, including a retrospective collection of corresponding clinical data detailing level of care, respiratory support and mode of feeding; however, no source was identified. It was established that none of the babies in putative transmission clusters were siblings, excluding the possibility of acquisition of parental flora by twins or triplets being interpreted incorrectly as transmission events. All individuals for whom an isolate was identified as part of a putative cluster had overlapping admission dates with other individuals within that cluster, providing evidence of an epidemiological link through shared exposure in space and time.

## Discussion

This evaluation of a single neonatal unit shows compelling evidence that transmission of bacteria between individuals resulting in subsequent colonization may be common within this setting. To our knowledge, this is the first study using WGS to elucidate the potential transmission dynamics of colonizing Gram-negative flora in the neonatal unit context. The majority of putative transmission clusters show a high level of similarity, suggesting that transmission is likely to have occurred recently. This most likely represents person-to-person transmission – likely on the hands of healthcare workers or other caregivers, or via fomites from their immediate environment e.g. medical devices, personal care equipment [22, 23]. The putative clusters in which isolates show a greater degree of divergence are less readily interpretable. Additional possibilities include a historical common source or maternal community colonization and subsequent mother-to-child transmission, although a common environmental source within the hospital environment cannot be excluded [24]. It is important to note that neonates cared for in the neonatal unit almost universally represent individuals who have never lived in the community. All babies who were implicated in the putative transmission clusters had overlapping admission dates, indicating an indirect epidemiological link due to a common location in space and time. Beyond this, we were not able to identify a specific transmission route (such as a shared medical device or feeding product) in a limited retrospective search. Factors that do not represent direct routes or reservoirs of transmission but which have been associated with outbreaks, such as nursing staff fluctuations, could not be assessed [25]. Nevertheless, we do feel that these findings support a continued focus on hand hygiene as a major route of nosocomial transmission. As sequencing capacity and expertise within the clinical realm continues to expand, it is interesting to reflect on whether near real-time availability of sequencing results would have enabled establishment of transmission routes with greater certainty, and afforded the opportunity to reduce or eliminate the transmission risk. A recent study of *Staphylococcus aureus* infection and colonization within a German neonatal unit similarly showed evidence of frequent transmission events occurring in spite of regular screening and enhanced infection prevention and control measures [26].

While some degree of AMR gene carriage was shown in all but one putative cluster, there was no evidence in this limited dataset of transmission of the most extensively drug-resistant isolates between individuals. As the routine screening only aimed to detect ESBL or gentamicin-resistant organisms, this transmission was not detected by the clinical laboratory. The detection of *bla*_CTX-M-15_-containing *K. pneumoniae* ST43 in isolates considered β-lactam sensitive after initial phenotypic screening is of uncertain significance; however, the relationship between the contexts of *bla*_CTX-M-15_ in these isolates and in a phenotypically β-lactam-resistant isolate of *E. coli* ST127 from the same individual is concerning, as it is possibly indicative of a horizontal gene transfer event. While chromosomal carriage of *bla*_CTX-M-15_ in uropathogenic *E. coli* ST127 has been described previously [27], the particular chromosomal context seen here appears to be unique, consistent with it having arisen in this setting. If transfer of this gene from *K. pneumoniae* ST43 to *E. coli* ST127 did in fact occur in this individual, the implication is that a phenotypically sensitive isolate carrying an ESBL-encoding resistance gene could transfer that gene to other organisms, rendering them β-lactam resistant. This has important implications for culture-dependent screening for β-lactam resistance in hospitals. While horizontal gene transfer is unproven in this case, it does highlight how the high resolution afforded by this data could help us to understand the development of AMR in individual cases and settings, with the potential to tailor infection prevention and control plans accordingly.

The infection prevention and control management of known ESBL-colonized babies demonstrates that awareness of a pathogen of concern did not always translate into isolation or cohort formation. This was likely due to a combination of limited isolation facilities, shortages of skilled specialist nursing staff and clinical need, meaning that babies could not always be safely isolated. These resource limitations highlight that even in high-income settings, there can remain significant impediments to instituting effective control measures. As WGS moves increasingly into the clinic, it will be important to consider how best to target this resource to maximize benefits to patients.

The unexpected finding of *Cronobacter* species that had not been identified by the clinical laboratory but were subsequently identified by WGS is not felt to be clinically significant within this dataset, as this organism is common in dried food sources and no associated invasive infections were seen. However, it is interesting to note that *Cronobacter* can be a significant foodborne source of infections and outbreaks in this age group, and, given the apparent difficulties in differentiation from *Enterobacter* species, it may be that a lower threshold for pursuing alternative means of identification should be considered if unexpected levels of invasive *Enterobacter* are seen in this population. This further points to the potential utility of WGS for surveillance of potential outbreaks over and above its role as a tool for investigation of suspected outbreaks.

The primary aim of this study was to uncover the Gram-negative genomic epidemiology within the neonatal unit, augmenting the results of standard culture-dependent screening, and ultimately to guide our understanding of the utility of Gram-negative screening in its current form in this patient group. WGS enabled a greater understanding of transmission dynamics, allowing us to reflect on how these results can benefit our patients. Our results, suggesting that transmission of Gram-negative organisms was likely to be widespread between individuals and that horizontal transfer of AMR genes was probable, demonstrate the limitations in culture-dependent screening of Gram-negative organisms, as well as emphasizing the importance of routine infection prevention and control measures – e.g. hand hygiene and equipment decontamination - to prevent nosocomial spread. The results may be generalizable to other neonatal units; however, it is important to note that this study looked at routine screening in the absence of any suspected outbreak. The use of Gram-negative screening during a suspected or confirmed outbreak is a very different context and is not evaluated here. Routine screening is resource intensive for the clinical laboratory, requiring significant skilled staff time, and effects on antimicrobial stewardship and development of AMR are largely unexplored. The paucity of evidence for benefit in directing specific infection control interventions and individualized empiric antibiotic management, as demonstrated here, and increasing pressures on the clinical laboratory workflow due to the ongoing COVID-19 pandemic, have led to the cessation of routine Gram-negative screening in this unit.

The use of WGS in this study helped us to understand possible transmission dynamics and inform our understanding of AMR in this context. Challenges remain in producing clinically actionable results, with barriers including lack of expertise in most clinical laboratories, speed and cost. Nevertheless, WGS data has the potential to identify transmission dynamics at a high level of acuity, and could be very helpful in the detection and investigation of outbreaks when used in a directed fashion, as described by Parcell *et al*. [28], and it would be helpful to see clinical studies focused on the best deployment strategies for WGS.

## Supplementary Data

Supplementary material 1
