# Supplementary material for: Whole-genome sequencing enhances existing pathogen and antimicrobial-resistance surveillance schemes within a neonatal unit
Source: Microb Genom. 2022 Jun 13;8(6):mgen000841. doi: 10.1099/mgen.0.000841 (PMC9455706; doi:10.1099/mgen.0.000841)
Supplement: Supplementary material 1 [file mgen-8-841-s001.pdf]

Supplementary Figure 1. Gram negative organism species identified by culture isolated from neonates admitted to the neonatal unit during the study period, presented by individual, site, and time (study week), with clinically important antimicrobial resistance phenotypes indicated. Isolates selected for sequencing were those identified as *E. coli, E. cloacae, K. pneumoniae* and *K. oxytoca*, as the 4 most prevalent species. Dates are presented on the X axis, individual participants are indicated on the y axis. R indicates a rectal or faecal sample, U indicates an umbilical sample. Abbreviations used: ECOL = *E. coli*, ECLC = *Enterobacter cloacae* complex, KPNE = *Klebsiella pneumoniae*, KOXY = *Klebsiella oxytoca*, CKOS = *Citrobacter koserii*, CITR = *Citrobacter* species, CFRE = *Citrobacter freundii*, CBRA = *Citrobacter braakii*, CYOU = *Citrobacter youngae*, CSAK = *Cronobacter sakazakii*, CMAL = *Cronobacter maloniticus,* KAER = *Klebsiella aerogenes*, KVAC = *Klebsiella variicola*, RPLA = *Raoultella planticola*, RORN = *Raoultella orthonylitica*, RSPE = *Raoultella* species, SLIQ = *Serratia liquefaciens*, SMAR = *Serratia marcescens*, ALWO = *Acinetobacter lwolfii*, PGER = *Pluralibacter gergoviae,* PMIR = *Proteus mirabilis*, COLI = unidentified coliform.

		Week 1: 2/8-9/8	Week 2: 10/8-16/8	Week 3: 17/8-23/8	Week 4: 24/8-30/8	Week 5: 31/8-6/9	Week 6: 7/9-13/9	Week 7: 14/9-20/9	Week 8: 21/9-27/9
1	R	KSPEC CKOS							
	U								
2	R	ECOL							
	U								
3	R	KOXY KOXY KOXY	KOXY KOXY	KOXY	KOXY		KOXY	KOXY KPNE SMAR	
	U	KOXY							
4	R	ECLC CITR CITR	ECLC CFRE	ECLC ECLC CFRE	ECLC CBRA				
	U				ECLC ECLC				
5	R	ECLC **KPNE** KPNE	KPNE **KAER**	ECLC **KPNE** KPNE	ECOL KPNE	**ECOL** **ECOL** **ECOL** KPNE	ECOL KPNE	**KPNE** **KPNE**	
	U		KAER						
6	R	ECOL ECOL							
	U								
7	R	**ECOL** **ECOL** ECLC ECLC							
	U								
8	R	ECOL	ECOL						
	U		ECOL						
9	R	ECLC RORN	RORN RPLAN	RORN	ECOL KPNE KOXY	ECOL ECOL KOXY KOXY			
	U	KOXY	RORN	KOXY RORN					
10	R	**ECLC**	**ECLC** **ECLC** KAER	ECLC			ECOL **KAER** **KAER**		ECOL KPNE
	U		**ECLC**			ECOL **ECLC** KAER KAER	ECOL **KAER**		KAER
11	R	ECLC KOXY KOXY CFRE							
	U	ECLC KOXY COLI							
12	R		KPNE	KPNE CFRE CFRE RSPE					
	U								
13	R		ECLC						
	U								
14	R		ECLC ECLC						
	U								
15	R		ECLC KOXY SLIQ	**ECOL** KOXY SLIQ	ECOL ECLC KOXY	ECOL ECLC KOXY SLIQ	ECOL KOXY		
	U					KOXY			
16	R		KOXY	KOXY KOXY	ECOL KOXY KOXY	ECOL KOXY PGER		ECOL KOXY	
	U								
17	R		ECOL	ECOL					
	U								
18	R		**ECLC** ECLC	ECLC KPNE	**ECOL** KPNE	**ECOL** ECLC KPNE			
	U								
19	R		ECOL ECOL						
	U		ECOL ECLC PMIR						
20	R			ECLC ECLC					
	U								
21	R			ECLC ECLC	CITR RSPE SMAR	KPNE SMAR SMAR SMAR	KPNE SMAR CYOU	KOXY SMAR	
	U							KOXY SMAR ALWO	
22	R			ECOL KPNE **KOXY** KOXY	KPNE KOXY KOXY				
	U			**ECLC** ECLC KOXY		KOXY KOXY			
23	R			ECOL	**ECOL** ECOL ECOL				
	U								
24	R			SLIQ	ECOL RORN RPLAN				
	U			SLIQ					
25	R			KPNE	ECLC KOXY				
	U								
26	R			**ECOL** **ECOL**					
	U			**ECOL**					
27	R				ECOL ECOL CKOS				
	U								
28	R				CSAK				
	U								
29	R				ECLC KPNE	ECLC KPNE KPNE			
	U								
30	R				ECLC ECLC ECLC				
	U								
31	R					ECOL **KPNE** **KPNE** **COLI**	ECOL KAER		
	U								
32	R					ECOL			
	U								
33	R					ECLC KOXY			
	U					ECLC KOXY			
34	R				ECOL				
	U								
35	R						KOXY	ECLC KOXY	
	U								
36	R							ECOL	
	U								
37	R							KAER	KAER
	U								
38	R							**KPNE** CMAL	**KPNE**
	U								
39	R							ECOL	ECOL KPNE
	U								KPNE
40	R							ECOL CKOS SMAR	
	U								
41	R							ECLC	ECLC
	U								
42	R								ECOL
	U								
43	R								KVAC
	U								
44	R								**KPNE**
	U								
45	R								KPNE
	U								
46	R								**KOXY** KOXY
	U								
47	R								**ECOL**
	U								
48	R								ECOL
	U								

Table showing screening isolates by individual over time

E coli
Enterobacter sp
Klebsiella oxytoca
Klebsiella pneumoniae
Other

NORMAL TEXT = no significant drug resistance seen (within limitations of testing already done)
BOLD TEXT = potentially significant drug resistance seen
Isolates have matching antibiograms
Isolates may have matching antibiograms, but data insufficient/lacking
Isolates have matching antibiograms which are distinct from any series marked with solid border

Supplementary Figure 2. Phylogenetic trees demonstrating relationships between isolates within the putative transmission clusters identified, generated using Parsnp. Only clusters with >2 isolates are included. Colour differentiation has been used to indicate isolates belonging to different individuals; SNP differences within the cluster are indicated in the sub-headings. Core genome sizes are: *E. coli* ST2562; 4,728,304 bp: *E. coli* ST429; 4,573,681 bp: *E. cloacae* ST631; 4,344,192 bp: *K. pneumoniae* ST1235; 5,490,822 bp: *K. oxytoca* Cluster 1; 5,477,628 bp: *K. oxytoca* Cluster 2; 5,197,084 bp.

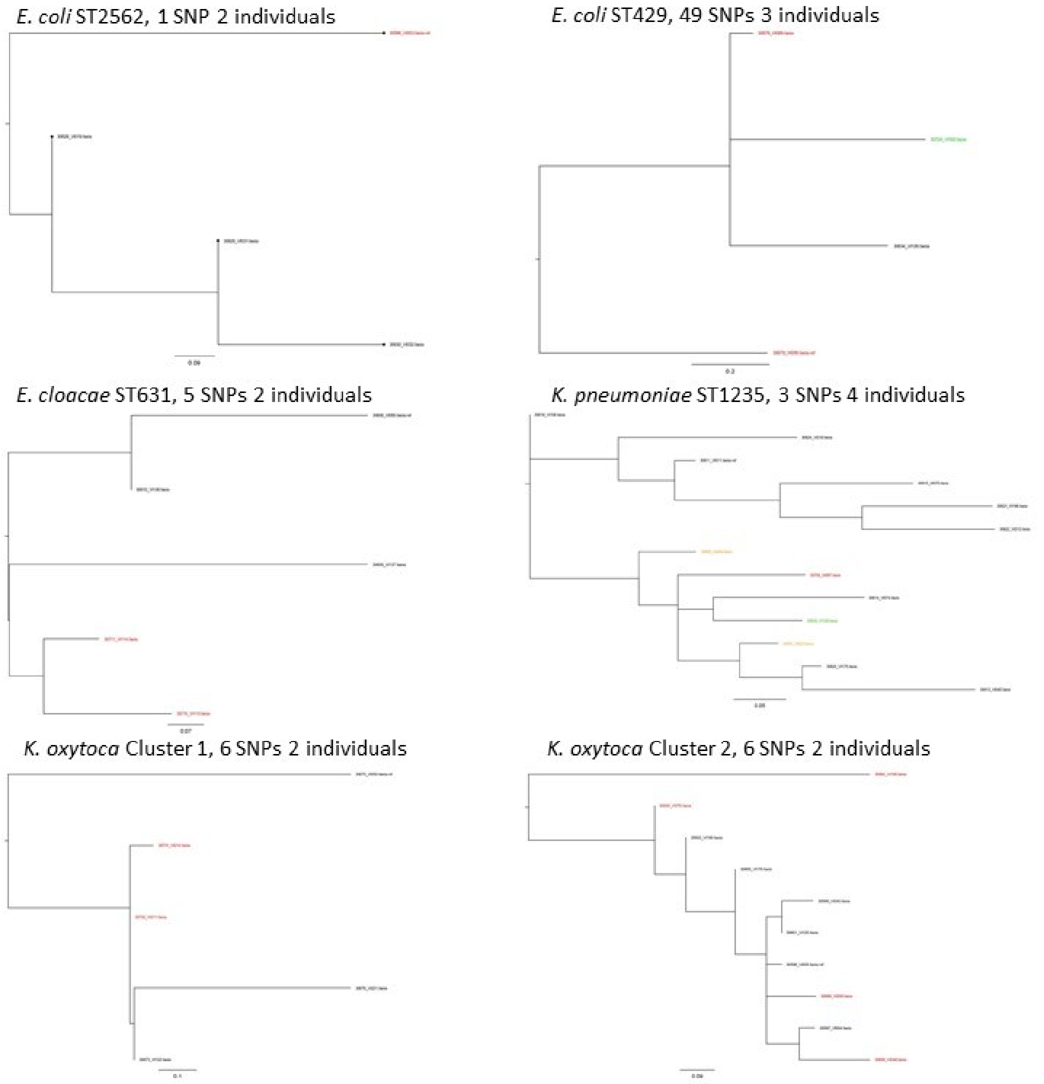

E. coli ST2562, 1 SNP 2 individuals

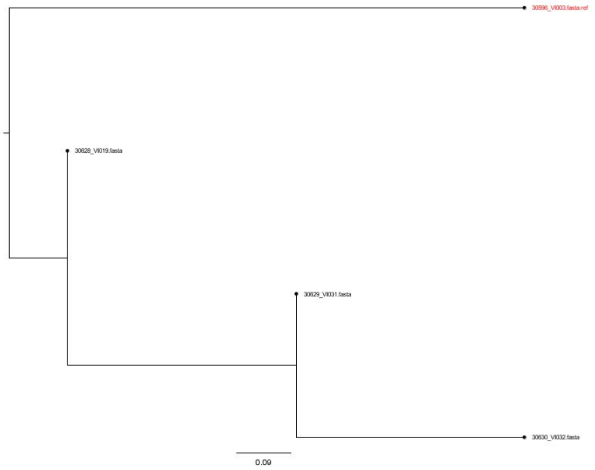

E. coli ST429, 49 SNPs 3 individuals

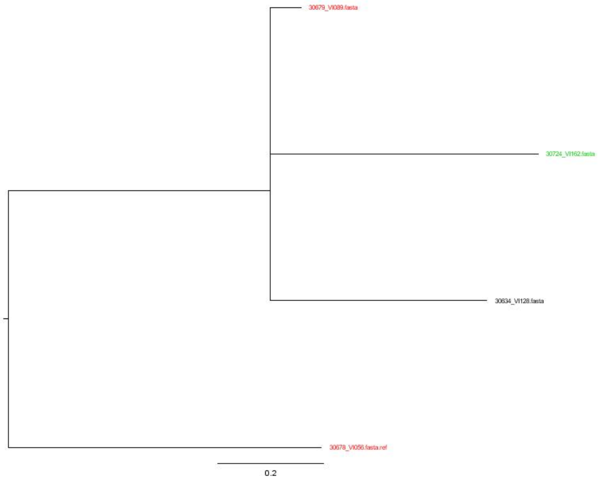

E. cloacae ST631, 5 SNPs 2 individuals

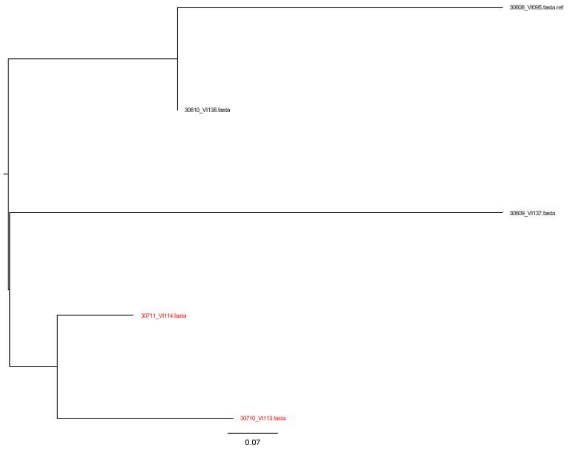

K. pneumoniae ST1235, 3 SNPs 4 individuals

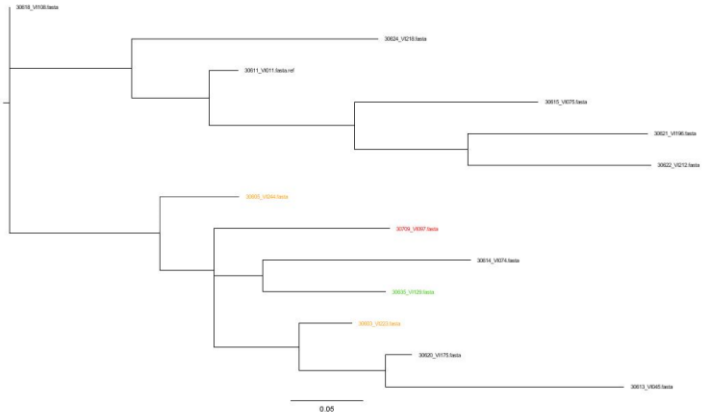

K. oxytoca Cluster 1, 6 SNPs 2 individuals

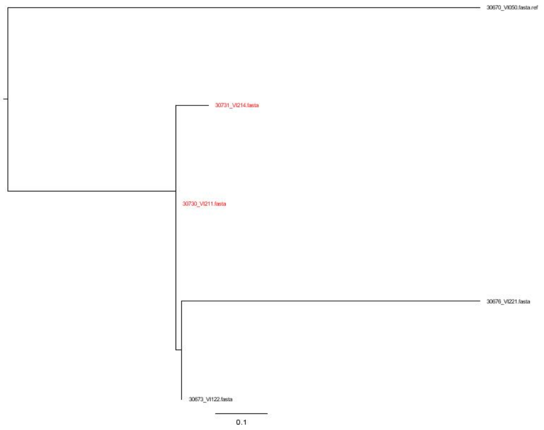

K. oxytoca Cluster 2, 6 SNPs 2 individuals

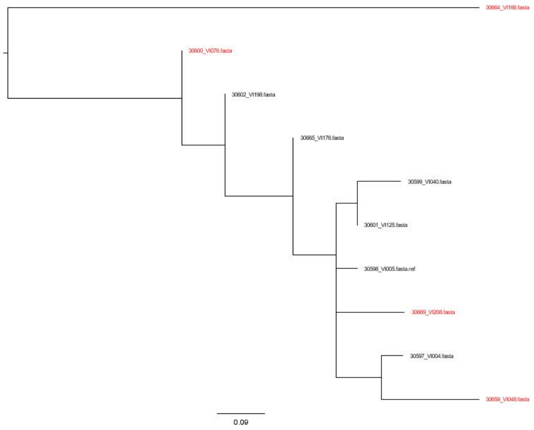

Supplementary table 1. GenBank sequences that contain the Tn*2*::IS*Ecp1*-*bla*$_{\text{CTX-M-15}}$ transposition unit configuration seen in *K. pneumoniae* STx isolate 191 (last search September 30, 2021)

Accession	Host	Country	Source	Year
CP010390	*K. pneumoniae*	Colombia	human bodily fluid	2012
CP028817	*K. pneumoniae*	Spain	human amniotic fluid	2012
CP012993	*K. pneumoniae*	Canada	human blood	2013
CP012988	*K. pneumoniae*	Canada	human urine	2013
CP016925	*K. pneumoniae*	USA	human tracheal aspirate	2014
CP074581	*K. pneumoniae*	India	human blood	2014
CP070465	*K. pneumoniae*	India	human blood	2014
CP072423	*K. pneumoniae*	India	human blood	2014
CP067256	*K. pneumoniae*	Switzerland	imported chilli pepper	2014
CP021166	*K. pneumoniae*	USA	human urine	2016
CP052364	*K. pneumoniae*	South Korea	human blood	2016
CP052193	*K. pneumoniae*	South Korea	human blood	2016
CP052263	*K. pneumoniae*	South Korea	human blood	2016
CP052307	*K. pneumoniae*	South Korea	human blood	2016
CP052730	*K. pneumoniae*	South Korea	human blood	2016
CP052599	*K. pneumoniae*	South Korea	human blood	2016
CP052566	*K. pneumoniae*	South Korea	human blood	2016
CP052441	*K. pneumoniae*	South Korea	human blood	2016
CP052374	*K. pneumoniae*	South Korea	human blood	2016
CP052358	*K. pneumoniae*	South Korea	human blood	2016
CP052151	*K. pneumoniae*	South Korea	human blood	2016
CP052140	*K. pneumoniae*	South Korea	human blood	2017
CP052338	*K. pneumoniae*	South Korea	human blood	2017
CP052391	*K. pneumoniae*	South Korea	human blood	2017
CP052243	*K. pneumoniae*	South Korea	human blood	2017
CP052330	*K. pneumoniae*	South Korea	human blood	2017

CP065168	*K. pneumoniae*	Australia	human urine	2017
CP065173	*K. pneumoniae*	Australia	human urine	2017
CP063014	*K. pneumoniae*	Russia	human urine	2017
CP056521	*K. pneumoniae*	UK	wastewater influent	2017
CP056435	*K. pneumoniae*	UK	wastewater influent	2017
AP021931	*K. pneumoniae*	Japan	wastewater effluent	2018
AP022554	*K. pneumoniae*	Japan	human urine	2018
CP079727	*K. pneumoniae*	China	human urine	2018
CP066535	*K. pneumoniae*	China	hospital environment	2019
CP082043	*K. pneumoniae*	China	human hydrothorax	2019
MH745930	*K. pneumoniae*	UK	-	-
MH745929	*K. pneumoniae*	UK	-	-
CP024516	*K. pneumoniae*	Australia*	-	-
CP024508	*K. pneumoniae*	Australia*	-	-
LR890572	*K. pneumoniae*	Australia*	-	-
LR890557	*K. pneumoniae*	Australia*	-	-
LR890485	*K. pneumoniae*	Australia*	-	-
LR890392	*K. pneumoniae*	Australia*	-	-
LR890385	*K. pneumoniae*	Australia*	human sputum	-
LR890241	*K. pneumoniae*	Australia*	-	-
LR890231	*K. pneumoniae*	Australia*	human rectal swab	-
LR890203	*K. pneumoniae*	Australia*	-	-
MW650887	*K. pneumoniae*	Italy	-	-
KT896504	*K. pneumoniae*	Israel*	wastewater	-
CP056583	*K. oxytoca*	UK	wastewater influent	2017
CP070022	*K. oxytoca*	USA	-	-
CP065164	*K. variicola*	Australia	human urine	2017
GU371928	*E. coli*	Belgium	human clinical	≤2010
CP003290	*E. coli*	USA	human clinical	2011
KM023153	*E. coli*	Nigeria	human faeces	≤2014
LR882051	*E. coli*	Norway*	wild bird	2016

AP022298	*E. coli*	Japan	oceanic water	2017
CP057511	*E. coli*	UK	sheep faeces	2017
MK965545	*E. coli*	Brazil	chicken meat	≤2019
CP026200	*E. coli*	USA	human	-
LR999867	*E. coli*	Finland*	barnacle goose faeces	-
LR999865	*E. coli*	Finland*	barnacle goose faeces	-
LT985221	*E. coli*	France*	-	-
CP040574	*S. enterica* Typhi	Denmark	human blood	2019
LT906492	*S. enterica* Typhi	UK*	human blood	-
LT882487	*S. enterica* Typhi	UK*	human blood	-
CP044008	*S. enterica* Typhi	USA*	-	-
CP071069	*P. stuartii*	Switzerland	human decubitus ulcer	2020

“-“ = data not included in GenBank entry

“*” = location data not present in GenBank entry, location of submitting author listed here
